# Peripheral precocious puberty in Li–Fraumeni syndrome: a case report and literature review of pure androgen-secreting adrenocortical tumors

**DOI:** 10.1186/s13256-023-03889-y

**Published:** 2023-05-14

**Authors:** Sofie Ryckx, Jean De Schepper, Philippe Giron, Ken Maes, Freya Vaeyens, Kaat Wilgenhof, Pierre Lefesvre, Caroline Ernst, Kim Vanderlinden, Daniel Klink, Frederik Hes, Jesse Vanbesien, Inge Gies, Willem Staels

**Affiliations:** 1 Division of Pediatric Endocrinology and Diabetology, Department of Pediatrics, ZNA Queen Paola Child Hospital, Lindendreef 1, 2020 Antwerp, Belgium; 2 Division of Pediatric Endocrinology, Department of Pediatrics, Vrije Universiteit Brussel (VUB), Universitair Ziekenhuis Brussel (UZ Brussel), Laarbeeklaan 101, 1090 Brussels, Belgium; 3 Centre for Medical Genetics, Vrije Universiteit Brussel (VUB), Universitair Ziekenhuis Brussel (UZ Brussel), Brussels, Belgium; 4 Department of Pathology, Vrije Universiteit Brussel (VUB), Universitair Ziekenhuis Brussel (UZ Brussel), Brussels, Belgium; 5 Department of Radiology, Vrije Universiteit Brussel (VUB), Universitair Ziekenhuis Brussel (UZ Brussel), Brussels, Belgium; 6 Division of Pediatric Surgery, Department of Surgery, Vrije Universiteit Brussel (VUB), Universitair Ziekenhuis Brussel (UZ Brussel), Brussels, Belgium; 7 Beta Cell Neogenesis (BENE) Research Group, Vrije Universiteit Brussel (VUB), Brussels, Belgium

**Keywords:** Pure androgen-secreting adrenocortical tumor, Li–Fraumeni syndrome, Peripheral precocious puberty, Pediatric endocrinology, Hypertension

## Abstract

**Introduction:**

Pure androgen-secreting adrenocortical tumors are a rare but important cause of peripheral precocious puberty.

**Case presentation:**

Here, we report a pure androgen-secreting adrenocortical tumor in a 2.5-year-old boy presenting with penile enlargement, pubic hair, frequent erections, and rapid linear growth. We confirmed the diagnosis through laboratory tests, medical imaging, and histology. Furthermore, genetic testing detected a pathogenic germline variant in the *TP53* gene, molecularly confirming underlying Li–Fraumeni syndrome.

**Discussion:**

Only 15 well-documented cases of pure androgen-secreting adrenocortical tumors have been reported so far. No clinical or imaging signs were identified to differentiate adenomas from carcinomas, and no other cases of Li–Fraumeni syndrome were diagnosed in the four patients that underwent genetic testing. However, diagnosing Li–Fraumeni syndrome is important as it implies a need for intensive tumor surveillance and avoidance of ionizing radiation.

**Conclusion:**

In this article, we emphasize the need to screen for *TP53* gene variants in children with androgen-producing adrenal adenomas and report an association with arterial hypertension.

**Supplementary Information:**

The online version contains supplementary material available at 10.1186/s13256-023-03889-y.

## Established facts and novel insights

Established facts

- Pure androgen-secreting adrenocortical tumors are a rare but important cause of peripheral precocious puberty.
- Molecular genetic analyses have become increasingly important in the workup of pediatric endocrine tumors, including adrenocortical malignancies.

Novel insights

- A pure androgen-secreting adrenocortical adenoma can be the first presentation of Li–Fraumeni syndrome.
- Androgen-secreting adrenocortical tumors can be associated with arterial hypertension

## Introduction

Peripheral precocious puberty (PPP), contrary to central precocious puberty (CPP), is caused by the autonomous secretion of androgens or human chorionic gonadotropin (hCG). Differentiating CPP from PPP in males is relatively straightforward and based on clinical and hormonal findings. In CPP, boys have testicular growth and elevated gonadotrophin levels, whereas in PPP, testicular volume is prepubertal and gonadotrophins are suppressed. However, diagnosing the cause of PPP can be more challenging, and includes neoplasms such as Leydig cell tumors, hCG-producing germ cell tumors, and androgen-secreting adrenocortical tumors (ACT) [1].

ACT are very rare in children; they can be benign or malignant, and their reported annual incidence is 0.2–0.3 new cases per million per year [2]. Contrary to adults, most ACT are functional in children and secrete cortisol, often together with androgens [3]. They are usually sporadic and benign but sometimes associated with tumor predisposition syndromes, such as Beckwith–Wiedemann syndrome and Li–Fraumeni syndrome (LFS) [4]. However, pure androgen-secreting adrenocortical tumors (PASACT) are ultra rare, and most reports describe solitary cases. We report a 2.5-year-old boy who presented with isosexual PPP due to a PASACT as the first expression of LFS, complicated by apparent tumor-related arterial hypertension. This case illustrates the differential diagnosis of PPP in young boys and highlights the importance of performing histopathological and genetic analysis upon diagnosing PASACT. We also reviewed previous reports of children with PASACT to identify common characteristics and potential clinical, imaging, or biochemical features that could allow preoperative differentiation between adrenocortical adenomas and carcinomas.

## Case presentation

**Fig. 1:**
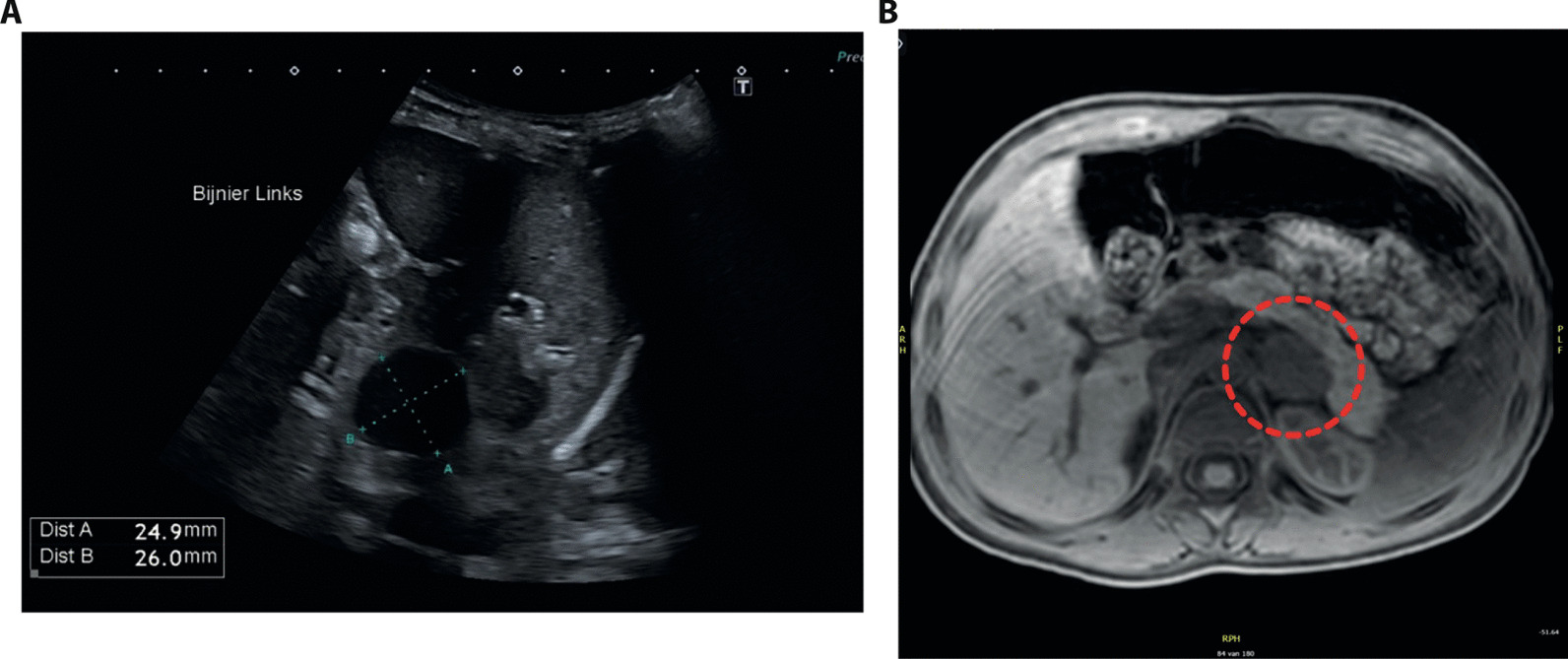
Imaging studies. **A** Abdominal ultrasound showed a hypoechoic lesion in the left adrenal. **B** MRI confirmed the presence of a well-defined 26 × 23 × 31 mm nodule (dotted red circle) with limited but homogeneous contrast accumulation, and no evidence of invasion into adjacent organs or vessels

A 2.5-year-old boy presented with a 6-month history of penile enlargement, pubic hair, frequent erections, and rapid linear growth. Delta height was + 1.19 standard deviation score (SDS) in the past 8 months. Previous medical and family history was unremarkable, and exposure to exogenous testosterone was excluded. On physical examination, his weight was 19.1 kg (+ 2.5 SD), height 101 cm (+ 1.7 SD), systolic blood pressure 138 mmHg (> 99th percentile), and diastolic 75 mmHg (95th–99th percentile). Tanner stage was A1 P4 G3, and penile length was 6.8 cm. He had symmetrical prepubertal testes (3 ml bilateral) without palpable abnormalities. Skin and oral examination were normal. Repeated blood pressure readings in the following weeks were all above the 95th percentile. His bone age was 3.5 years, as per the Greulich–Pyle scale. Serum gonadotrophin levels were prepubertal, while testosterone (9.9 µg/L, ref. < 0.12 µg/L), androstenedione (2365 ng/l, ref. 100–900 ng/L), and dehydroepiandrosterone-sulfate (DHEA-S) levels (1.49 mg/L, ref. < 0.02–0.15 mg/L) were elevated, approaching the adult reference range. Serum electrolytes, 17-OH-progesterone, adrenocorticotropic hormone (ACTH), renin, aldosterone, and cortisol levels were normal, as were repetitive 24-hour urinary free cortisol excretion and urinary catecholamine levels (Table 1). Testicular ultrasound was normal. Abdominal ultrasound showed a hypoechogenic ovoid nodule in the left adrenal (Fig. 1A), which on magnetic resonance imaging (MRI) was well defined (26 × 23 × 31 mm) and hyperintense on T2 weighted sequences. Contrast accumulation was limited and homogeneous, and there was no evidence of invasion into adjacent organs or vessels (Fig. 1B). Chest X-ray and liver ultrasound imaging were normal. We decided on laparoscopic unilateral adrenalectomy on the basis of the hormonal findings and imaging features. Two days after surgery, blood pressure normalized, and normal prepubertal levels of testosterone, androstenedione, and DHEA-S were found.

**Table 1:** Hormone levels before and after left adrenalectomy

	Before surgery	1 month after surgery	References
LH (IU/L)	< 1.0	0.5	0.10–1.29
FSH (IU/L)	< 1.0	1.7	0.21–2.8
ACTH (ng/L)	24.1	24.4	8–10 hours: 7.2–63
IGF1-1 (µg/L)	270	156	23–212
Cortisol (µg/L)	70.4	78.1	7–10 hours: 62–180
DHEA-S (mg/L)	1.49	0.08	< 0.02–0.15
Estradiol (ng/L)	9.9	< 5	< 20
Androstenedione (ng/L)	2365	53	100–900
Testosterone (µg/L)	9.90	< 0.12	< 0.12
SHBG (nmol/L)	62.2	92.5	42.4–155.6

*LH* luteinizing hormone; *FSH* follicule stimulating hormone; *ACTH* adrenocorticotropic hormone, *IGF-1* insulin-like growth factor 1; *DHEA-S* dehydroepiandrosterone-sulfate, *SHBG* sex hormone binding globulin

**Fig. 2:**
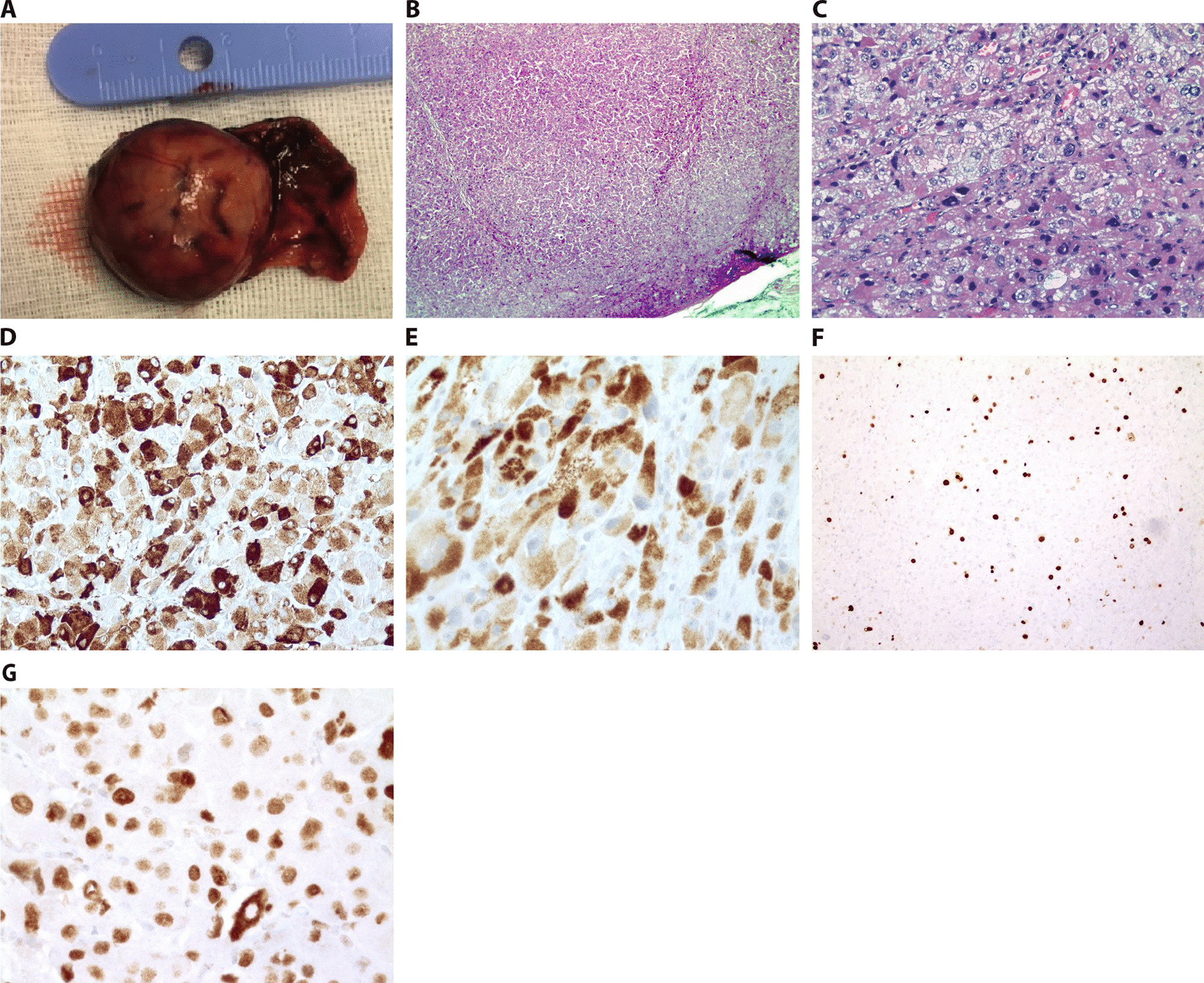
Pathology. **A** Macroscopic image of the adrenal tumor with an intact thin capsule. Weight 11.2 g (after fixation), dimensions 4.5 cm × 3 × 2.5 cm, and maximum diameter 2.5 cm **B** Low magnification and **C** high magnification image of H&E staining showing a solid population of eosinophilic cells with granular cytoplasm, vesicular nuclei with moderate pleomorphism, and some giant nuclei. No hemorrhagic or necrotic zones. **D**, **E** Positive immunostaining for (**D**) inhibin and (**E**) Melan-A. **F** Low mitotic activity with a Ki67 positive cell ratio of approximately 10%. **G** Strong and diffuse P53 immunoexpression (100% of positive cells)

Macroscopically, the tumor was well defined and surrounded by a thin capsule (Fig. 2A). Histologically it contained areas of nuclear pleomorphism but no necrosis (Fig. 2B, C). There were no signs of capsular or vascular invasion. The tumor stained positive for inhibin (Fig. 2D) and Melan-A (Fig. 2E). It was classified as a cortical neoplasm with a favorable prognosis on the basis of (immuno)histological characteristics (Wieneke criteria, score: 0): tumor weight < 200 g, no capsular invasion or tumor necrosis, mitotic activity < 15/20 high-power field (HPF), Ki67 index < 15% (Fig. 2F), and strong and diffuse P53 expression (3 + intensity, 100% of positive cells) (Fig. 2G). The comparative genomic hybridization of the tumor revealed a complex karyotype with multiple trisomies (Chr 1, 5, 7, 8, 9, 14, 16, and 19), monosomies (Chr 2, 3, 4, 10, 11, 17, and 21), and loss of chromosomes X and Y.

**Fig. 3:**
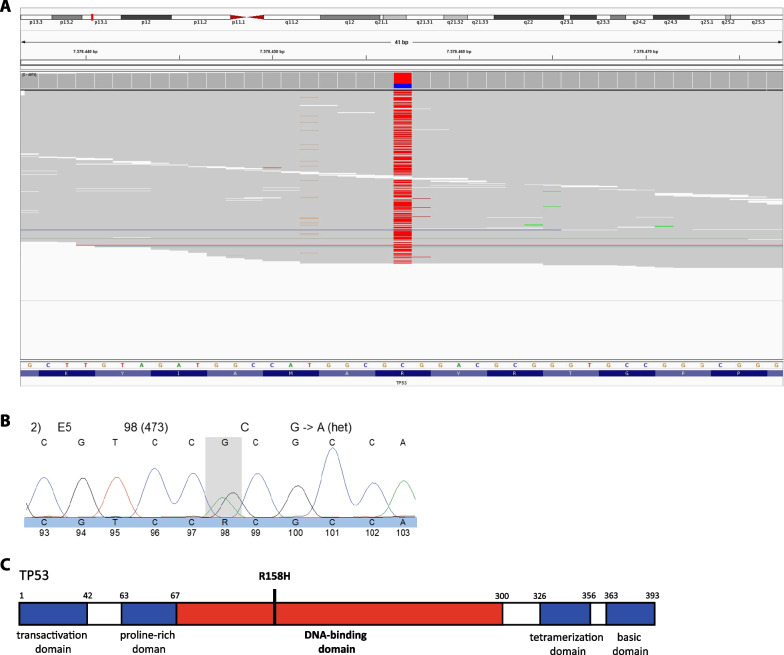
Genetic testing. **A** Integrative Genomics Viewer (IGV) graphic showing a single nucleotide variant (SNV) in the *TP53* gene (G>A), producing p.Arg158His alteration. The reference sequence used is NM_000546.5. **B** *TP53* gene diagram shows that the R158H variant hits the functional part of the DNA-binding domain of P53 and imparts a transcriptional activity comparable to null variants. **C** Domain structure of p53. Adapted from Tanaka *et al*. [40]

Massive parallel sequencing was performed on the tumor, analyzing a total of 165 cancer-related genes (additional file 1: Table S1), which identified a pathogenic *TP53* gene variant NM_0000546.5(TP53):c.473G > A, p.(Arg158His) (conform ClinVar database https://www.ncbi.nlm.nih.gov/clinvar), at an allelic frequency of 71%. Targeted Sanger sequencing on DNA from a whole blood sample subsequently confirmed the heterozygous germline presence of this pathogenic *TP53*-variant, confirming the diagnosis of LFS (see also Fig. 3A–C). Familial screening of the proband’s parents and brother was negative, indicating *de novo* occurrence of the variant. Two years after the adrenalectomy, the patient is in good health, and whole-body MRI cancer screening remains negative for abnormalities. Of note, there was no regression of the penis development or pubic hair.

## Case series

We conducted a literature search in Pubmed using the query “androgen AND adrenal AND pediatric AND (carcinoma OR adenoma)”. From the 76 retrieved articles, we selected 7 reports with detailed clinical and histological data. Finally, we retrieved 16 detailed case reports using citation searching. We summarized their clinical and histological findings, and when available, the results of genetic testing in Table 2 and compared them with our patient [5–19].

**Table 2:** Childhood PASACT case report summary

	First author, year (ref.)	Age at diagnosis	Sex	Signs and symptoms	Tanner stage	Testosterone (nmol/L)	Tumor size	Laterality	Invasion in surrounding tissue	Adenoma or carcinoma	Genetics/Ki67 index/histological grading
1	Burr, 1973 [5]	1 year 8 months	F	Increased labial size, labial hair	A1P3M1	1.49 (elevated)	2 × 1.5 × 0.5 cm	L	No	NR	NR
2	Richards, 1983 [6]	2 years 0 months	F	Deepening voice, acne, labial hair	A1P2M1	14.11 (< 0.69)	3.5 cm	R	No	A	NR
3	Kamilaris, 1987[7]	15 years	F	Primary amenorrhea, facial hair, acne, deepening voice	P4M1	12.2 (0.69–3.47)	2 × 2.5 cm	R	No	A	NR
4	Sorgo, 1988 [8]	12 years 10 months	F	Deepening voice	P4M5	7.28 (0.35–2.43)	1 × 2 cm	L	No	C	NR
5	Muenstere, 2001 [10]	6 years	M	Growth acceleration, gynecomastia, pubic hair	P3G3	16.02 (< 0.69)	2 cm	L	No	A	NR
6	Bavdekar, 2001 [9]	11 months	F	Pubic hair	A1P2M1	9.71 (0.52–3.29)	4.4 × 4.6 cm	R	No	C	NR
7	Valerio, 2003 [11]	4 years 4 months	M	Pubic hair, penile growth	A1P2G3	3.81 (< 0.69)	40 g	L	NR	A	NR
8	Miyoshi, 2009 [12]	6 years 6 months	M	Facial acne, growth acceleration, facial acne	A1P3G3	7.56 (elevated)	5.5 × 4.1 cm	R	No	C	No *TP53* mutation/Ki67 8%/Weisse 4
9	Kumar, 2010 [13]	3 years	M	Penis growth, pubic hair, deepening voice, axillary hair	A2P2G3	33.63 (0.42–1.11)	6.8 × 4.7 cm	L	No	C	Genetics NR/Ki67 NR/Weisse high grade
10	Choukair, 2013 [14]	2 years 4 months	F	Pubic hair, clitoral hypertrophy, growth acceleration	A1P3M1	7.08 (< 0.69)	1.3 cm L ectopic at renal hilus and 0.5 cm R adrenal	L + R	No	A	Genetics NR/Ki67 2 to 5%/Grade NR
11	Rodriguez-Gutiérrez, 2013 [15]	18 years	F	Hirsutism, clitoral hypertrophy, deepening voice, primary amenorrhea	A2P6M1	15.01 (0.21–2.84)	10 × 11 cm	L	No	A	NR
12	Marret, 2014 [17]	2 months	F	Pubic hair, growth acceleration	A1P3M1	1.66 (< 0.17)	1.7 × 0.9 × 1.1 cm	R	No	Unclear	No *TP53* mutation
13	Kim, 2015 [16]	8 years 2 months	M	Penis growth, pubic hair, facial acne	P2G3	13.24 elevated	7.8 × 6.1 × 4.0 cm	R	No	C	No *TP53* mutation/Ki67 30%/Weisse 6
14	Ersoy, 2017 [18]	4 years	F	Pubic hair, facial acne	P2M2	2.33 (< 0.026)	5.5 × 4 cm	L	No	A	NR
15	Kafi, 2019 [19]	4 years	M	Penis growth, pubic hair, facial acne	P3G3	Not mentioned	Not mentioned	R	NR	NR	NR
16	Proband (present study)	2 years 7 months	M	Penis growth, erections, pubic hair, growth acceleration	A1P4G3	29.1 (< 0.01)	2.6 × 2.3 × 3.1 cm	L	No	A	Heterozygous *TP53* mutation/Ki67 < 15%/Wieneke 0

*F* female; *M* male; *A* adrenarche; *P* pubarche; *M* mamma; *G* genital; *L* left; *R* right; *A* adenoma; *C* carcinoma; *NR* not reported

The median age at diagnosis was 4 years (range: 2 months–18 years), and there was a female sex bias (7 males versus 9 females). All patients had clinical signs of androgen excess, such as pubic hair before 8 years of age or voice deepening in five cases. In 5/7 male cases, patients had rapid or early penile growth, while clitoral hypertrophy was reported in just 2/9 female cases. In 6/16 cases, there was facial acne, and a single subject (a 3-year-old boy) had precocious axillary hair development. In 2/3 female patients older than 12 years (12 years 10 months, 15 years, and 18 years), there was primary amenorrhea, but one adolescent girl had regular menses.

Testosterone levels were elevated in all patients, and all investigated children had normal serum cortisol, 24-hour urinary free cortisol, and plasma adrenocorticotropic hormone levels (Table 2). The commonly used imaging modalities were abdominal ultrasound, CT, or MRI. Tumor laterality was symmetrical (9 right versus 8 left adrenal tumors). However, in one case the intraadrenal adenoma on the right side was accompanied by an ectopic adrenal adenoma in the left renal hilus. The median tumor size was 3.05 cm in diameter (< 1–11 cm). Eight tumors were histologically classified as adenoma, five as carcinoma, and four were not specified. All adenomas were smaller than 5 cm except for two cases. Cases of adenoma or carcinoma did not differ in age at presentation, sex distribution, presenting symptoms, tumor size, or laterality. Vascular or soft tissue invasion was absent in both adenomas and carcinomas in all 17 reported tumors in the 16 reported cases. None of the reported cases presented metastatic disease. Genetic testing for germline *TP53* gene variants was performed in 4/16 patients. Here, to the best of our knowledge, we report for the first time an underlying pathogenic *TP53* variant in childhood PASACT.

## Discussion

Penile growth without testicular enlargement in boys under 9 years of age is a typical sign of PPP. The differential diagnosis of PPP in boys is broad and includes exposure to exogenous testosterone, testotoxicosis, and non-classical congenital adrenal hyperplasia, but also neoplastic causes, including Leydig cell tumors, hCG-producing germ cell tumors, and androgen-secreting ACT [20]. Therefore, initial screening should include bone age assessment and measurement of gonadotropins, testosterone, hCG, DHEA-S, and 17-OH-progesterone levels. In the case of mainly elevated serum testosterone and androstenedione concentrations—as in this report—either a testicular or adrenal tumor should be sought. Indeed, in this case, we tentatively diagnosed a PASACT on the basis of clinical (well-defined nodule in the left adrenal gland with lack of invasion of adjacent tissue on MRI imaging and a normal testicular sonogram) and biochemical (high androgens and normal renin-aldosterone blood serum levels and normal urinary cortisol excretion) features.

In case of isolated hypercorticism and negative adrenal imaging, further workup with a dexamethasone test is warranted. However, in this case of PPP there were no signs of hypercorticism and imaging revealed the adrenal tumor. Therefore, no dexamethasone suppression test was carried out.

The subsequent histological analysis could confirm the diagnosis, and genetic testing demonstrated an underlying pathogenic *TP53* variant associated with LFS.

PASACT is a rare neoplastic cause of PPP that is either benign or malignant and most often presents under the age of 5 years [21, 22]. The initial treatment step for PASACT is surgical tumor resection, which we performed by unilateral laparoscopic adrenalectomy. However, the current literature shows that tumor size or soft tissue invasion cannot differentiate adenomas from carcinomas reliably. This implies that clinical signs and imaging cannot differentiate benign from malignant PASACTs. However, after surgery, histological tumor features such as higher mitotic rate, higher percentage of necrosis, and larger tumor size are more associated with carcinomas [23].

In the present case, the histology (low mitotic rate, no necrosis) and the normalization of androgen levels after surgery confirmed the diagnosis of a pure androgen-secreting adrenal adenoma. However, the histology of ACT in children is challenging, as the classical Weiss criteria used in adults often misclassify childhood ACT as carcinomas [24]. Therefore, a distinct pediatric grading system, the Wieneke criteria, should be combined with the Ki67 index and p53 status for childhood ACT [25–27]. Here, in the present case, both the Wieneke criteria (score: 0) and the Ki67 index (< 15%) indicated an adrenal adenoma. However, p53 status (100% 3 + nuclear p53 expression) suggested a *TP53*-mutation. Among the previously reported PASACT cases, only five mentioned the Ki67 index or the Weiss criteria, and none reported using the appropriate Wieneke criteria.

Both benign and malignant ACTs are reported in genetic tumor syndromes, such as Beckwith–Wiedemann syndrome, Carney complex, and LFS. In LFS, adrenal carcinomas have mainly been reported. In our case, there were no clinical signs of a tumor predisposition syndrome, and the family history was negative for tumors. Germline pathogenic variants in *TP53* are found in approximately 45–80% of children with adrenocortical carcinomas and a negative family history of LFS [28, 29]. Moreover, in up to 20% of LFS families, the *TP53* variants arose *de novo*. Hence, with its variable expression and penetrance, families with LFS are prone to be missed [30]. Nonetheless, germline *TP53* variants predisposing to LFS are found in more than half of children with ACT, but the majority were cortisol-secreting [31]. The tumor was characterized as an adenoma; however, it is unclear whether adrenocortical carcinomas arise as such or if they can originate from benign lesions such as hyperplasias or adenomas [32]. Therefore, we performed a *TP53* gene analysis in the proband. The specific *TP53* missense variant [NM_000546.5(TP53):c.473G > A] found in the presented case has been reported multiple times, and the resulting p.R158 substitution hits a functional part of the TP53 DNA-binding domain (Fig. 3C). Varley *et al*. were one of the first to describe this germline variant in 3/14 subjects in a study on childhood ACTs [28]. In a larger study regarding *TP53* gene variant carriers in LFS, Bougeard *et al*. reported that out of the 11 carriers of the p.Arg158His variant (from 8 different families), 4 were positive for a history of childhood adrenocortical carcinoma (ACC) [33]. Furthermore, this variant was the most identified germline alteration in 34 children with ACC [33].

It is important to identify the underlying type of *TP53* variant for clinical follow-up. Missense variants, including p.Arg158His, differ from null variants as patients with missense variants present approximately 9 years earlier than those with other *TP53* variants, including null mutations, suggesting an additional oncogenic effect in *TP53* missense variants [29]. Correspondingly, in a simplified p53 functional assay to quantify the induction of p53 transcriptional activity in response to DNA damage after exposure to doxorubicin, the mean functionality score in the p.Arg158His variant was about 50% of the mean score of the control group. This 50% reduction in functionality was like the remaining function in TP53 null variants, indicating a dominant negative effect [34]. Thus, intensive tumor surveillance with annual total body and brain MRI is needed in these patients, and exposure to ionizing radiation should be avoided. Furthermore, long-term endocrine follow-up is indicated as gonadotropin-dependent pubertal disorders are common in children with a history of virilizing ACT [35].

The arterial hypertension in the present case posed an additional diagnostic challenge, since both an excess of circulating secreted adrenal hormones and their metabolites or renal artery compression by a large ACT, with concomitant secondary hyperaldosteronism, were possible. Urinary cortisol excretion, renin–aldosterone state, and normal urinary catecholamines were normal, and renal artery compression was not detected. Furthermore, the rapidly normalized blood pressure after tumor resection strongly suggests a tumor-driven mechanism. Androgens have a blood-pressure-elevating effect. This has been studied in both animal models and humans. Blood pressure decreased after castrating male rats and increased after administration of testosterone both in normotensive female and in normotensive male rats [36]. Similarly, in transgender population studies, testosterone increased blood pressure [37]. An association of male sex steroids with the reduction of pressure natriuresis has been proposed previously as a contributing cause of hypertension [15]. In our review of childhood PASACT cases, only one patient had arterial hypertension, which normalized 2 months after surgery [15]. In this case, the levels of 11-deoxycorticosterone, a potent mineralocorticoid, were normal. However, mineralocorticoid receptor stimulation and subsequent hypertension by adrenal steroids that are not measured routinely cannot be excluded and might be involved in some PASACT cases [38, 39].

## Conclusion

Androgen-secreting tumors should be part of differential diagnosis in children with PPP. Clinical, biochemical, and imaging features are important for diagnosing an androgen-secreting ACT, but cannot differentiate benign from malignant tumors. Molecular genetic testing is important to ascertain whether an ACT is related to a specific genetic tumor predisposition syndrome, including LFS, even when histologically considered as having a favorable prognosis. Arterial hypertension can be associated with pure androgen-secreting adrenocortical adenomas in children.

## Supplementary Information

**Additional file 1: Table S1. **List of 165 analyzed cancer-related genes.

## Data Availability

All data generated or analysed during this study are included in this published article and its supplementary information files.
