# Supplementary material for: Peripheral precocious puberty in Li–Fraumeni syndrome: a case report and literature review of pure androgen-secreting adrenocortical tumors
Source: J Med Case Rep. 2023 May 14;17:195. doi: 10.1186/s13256-023-03889-y (PMC10183130; doi:10.1186/s13256-023-03889-y)
Supplement: Supplementary file 1 — Additional file 1: Table S1. List of 165 analyzed cancer-related genes. [file 13256_2023_3889_MOESM1_ESM.docx]

**Supplementary Table 1:** List of 165 analyzed cancer related genes.

| **Gene symbol** | **Full name (HGNC)** |
| --- | --- |
| ABL1 | ABL proto-oncogene 1, non-receptor tyrosine kinase [Source:HGNC Symbol;Acc:HGNC:76] |
| ABRAXAS1 | abraxas 1, BRCA1 A complex subunit [Source:HGNC Symbol;Acc:HGNC:25829] |
| ACVR1 | activin A receptor type 1 [Source:HGNC Symbol;Acc:HGNC:171] |
| AKT1 | AKT serine/threonine kinase 1 [Source:HGNC Symbol;Acc:HGNC:391] |
| ALK | ALK receptor tyrosine kinase [Source:HGNC Symbol;Acc:HGNC:427] |
| APC | APC regulator of WNT signaling pathway [Source:HGNC Symbol;Acc:HGNC:583] |
| AR | androgen receptor [Source:HGNC Symbol;Acc:HGNC:644] |
| ARAF | A-Raf proto-oncogene, serine/threonine kinase [Source:HGNC Symbol;Acc:HGNC:646] |
| ARID1A | AT-rich interaction domain 1A [Source:HGNC Symbol;Acc:HGNC:11110] |
| ASXL1 | ASXL transcriptional regulator 1 [Source:HGNC Symbol;Acc:HGNC:18318] |
| ATM | ATM serine/threonine kinase [Source:HGNC Symbol;Acc:HGNC:795] |
| ATR | ATR serine/threonine kinase [Source:HGNC Symbol;Acc:HGNC:882] |
| ATRX | ATRX chromatin remodeler [Source:HGNC Symbol;Acc:HGNC:886] |
| AXIN1 | axin 1 [Source:HGNC Symbol;Acc:HGNC:903] |
| BAP1 | BRCA1 associated protein 1 [Source:HGNC Symbol;Acc:HGNC:950] |
| BARD1 | BRCA1 associated RING domain 1 [Source:HGNC Symbol;Acc:HGNC:952] |
| BCL2 | BCL2 apoptosis regulator [Source:HGNC Symbol;Acc:HGNC:990] |
| BCL6 | BCL6 transcription repressor [Source:HGNC Symbol;Acc:HGNC:1001] |
| BCOR | BCL6 corepressor [Source:HGNC Symbol;Acc:HGNC:20893] |
| BRAF | B-Raf proto-oncogene, serine/threonine kinase [Source:HGNC Symbol;Acc:HGNC:1097] |
| BRCA1 | BRCA1 DNA repair associated [Source:HGNC Symbol;Acc:HGNC:1100] |
| BRCA2 | BRCA2 DNA repair associated [Source:HGNC Symbol;Acc:HGNC:1101] |
| BRIP1 | BRCA1 interacting helicase 1 [Source:HGNC Symbol;Acc:HGNC:20473] |
| BTK | Bruton tyrosine kinase [Source:HGNC Symbol;Acc:HGNC:1133] |
| CALR | calreticulin [Source:HGNC Symbol;Acc:HGNC:1455] |
| CARD11 | caspase recruitment domain family member 11 [Source:HGNC Symbol;Acc:HGNC:16393] |
| CBL | Cbl proto-oncogene [Source:HGNC Symbol;Acc:HGNC:1541] |
| CCND1 | cyclin D1 [Source:HGNC Symbol;Acc:HGNC:1582] |
| CD79B | CD79b molecule [Source:HGNC Symbol;Acc:HGNC:1699] |
| CDH1 | cadherin 1 [Source:HGNC Symbol;Acc:HGNC:1748] |
| CDK12 | cyclin dependent kinase 12 [Source:HGNC Symbol;Acc:HGNC:24224] |
| CDKN2A | cyclin dependent kinase inhibitor 2A [Source:HGNC Symbol;Acc:HGNC:1787] |
| CDKN2B | cyclin dependent kinase inhibitor 2B [Source:HGNC Symbol;Acc:HGNC:1788] |
| CDKN2C | cyclin dependent kinase inhibitor 2C [Source:HGNC Symbol;Acc:HGNC:1789] |
| CEBPA | CCAAT enhancer binding protein alpha [Source:HGNC Symbol;Acc:HGNC:1833] |
| CHEK1 | checkpoint kinase 1 [Source:HGNC Symbol;Acc:HGNC:1925] |
| CHEK2 | checkpoint kinase 2 [Source:HGNC Symbol;Acc:HGNC:16627] |
| CIC | capicua transcriptional repressor [Source:HGNC Symbol;Acc:HGNC:14214] |
| CRBN | cereblon [Source:HGNC Symbol;Acc:HGNC:30185] |
| CREBBP | CREB binding protein [Source:HGNC Symbol;Acc:HGNC:2348] |
| CSF3R | colony stimulating factor 3 receptor [Source:HGNC Symbol;Acc:HGNC:2439] |
| CTNNB1 | catenin beta 1 [Source:HGNC Symbol;Acc:HGNC:2514] |
| CUL4B | cullin 4B [Source:HGNC Symbol;Acc:HGNC:2555] |
| CXCR4 | C-X-C motif chemokine receptor 4 [Source:HGNC Symbol;Acc:HGNC:2561] |
| CYLD | CYLD lysine 63 deubiquitinase [Source:HGNC Symbol;Acc:HGNC:2584] |
| DAXX | death domain associated protein [Source:HGNC Symbol;Acc:HGNC:2681] |
| DDR2 | discoidin domain receptor tyrosine kinase 2 [Source:HGNC Symbol;Acc:HGNC:2731] |
| DICER1 | dicer 1, ribonuclease III [Source:HGNC Symbol;Acc:HGNC:17098] |
| DIS3 | DIS3 homolog, exosome endoribonuclease and 3'-5' exoribonuclease [Source:HGNC Symbol;Acc:HGNC:20604] |
| DNMT3A | DNA methyltransferase 3 alpha [Source:HGNC Symbol;Acc:HGNC:2978] |
| EGFR | epidermal growth factor receptor [Source:HGNC Symbol;Acc:HGNC:3236] |
| EGR1 | early growth response 1 [Source:HGNC Symbol;Acc:HGNC:3238] |
| EIF1AX | eukaryotic translation initiation factor 1A X-linked [Source:HGNC Symbol;Acc:HGNC:3250] |
| EP300 | E1A binding protein p300 [Source:HGNC Symbol;Acc:HGNC:3373] |
| EPCAM | epithelial cell adhesion molecule [Source:HGNC Symbol;Acc:HGNC:11529] |
| ERBB2 | erb-b2 receptor tyrosine kinase 2 [Source:HGNC Symbol;Acc:HGNC:3430] |
| ERBB3 | erb-b2 receptor tyrosine kinase 3 [Source:HGNC Symbol;Acc:HGNC:3431] |
| ERBB4 | erb-b2 receptor tyrosine kinase 4 [Source:HGNC Symbol;Acc:HGNC:3432] |
| ESR1 | estrogen receptor 1 [Source:HGNC Symbol;Acc:HGNC:3467] |
| ETV6 | ETS variant transcription factor 6 [Source:HGNC Symbol;Acc:HGNC:3495] |
| EZH2 | enhancer of zeste 2 polycomb repressive complex 2 subunit [Source:HGNC Symbol;Acc:HGNC:3527] |
| FANCA | FA complementation group A [Source:HGNC Symbol;Acc:HGNC:3582] |
| FANCL | FA complementation group L [Source:HGNC Symbol;Acc:HGNC:20748] |
| FAU | FAU ubiquitin like and ribosomal protein S30 fusion [Source:HGNC Symbol;Acc:HGNC:3597] |
| FBXW7 | F-box and WD repeat domain containing 7 [Source:HGNC Symbol;Acc:HGNC:16712] |
| FGFR1 | fibroblast growth factor receptor 1 [Source:HGNC Symbol;Acc:HGNC:3688] |
| FGFR2 | fibroblast growth factor receptor 2 [Source:HGNC Symbol;Acc:HGNC:3689] |
| FGFR3 | fibroblast growth factor receptor 3 [Source:HGNC Symbol;Acc:HGNC:3690] |
| FLT3 | fms related receptor tyrosine kinase 3 [Source:HGNC Symbol;Acc:HGNC:3765] |
| FOXL2 | forkhead box L2 [Source:HGNC Symbol;Acc:HGNC:1092] |
| FOXO1 | forkhead box O1 [Source:HGNC Symbol;Acc:HGNC:3819] |
| FUBP1 | far upstream element binding protein 1 [Source:HGNC Symbol;Acc:HGNC:4004] |
| GNA11 | G protein subunit alpha 11 [Source:HGNC Symbol;Acc:HGNC:4379] |
| GNAQ | G protein subunit alpha q [Source:HGNC Symbol;Acc:HGNC:4390] |
| GNAS | GNAS complex locus [Source:HGNC Symbol;Acc:HGNC:4392] |
| H1-4 | H1.4 linker histone, cluster member [Source:HGNC Symbol;Acc:HGNC:4718] |
| H3-3A | H3.3 histone A [Source:HGNC Symbol;Acc:HGNC:4764] |
| H3-3B | H3.3 histone B [Source:HGNC Symbol;Acc:HGNC:4765] |
| H3C2 | H3 clustered histone 2 [Source:HGNC Symbol;Acc:HGNC:4776] |
| H3C3 | H3 clustered histone 3 [Source:HGNC Symbol;Acc:HGNC:4768] |
| HRAS | HRas proto-oncogene, GTPase [Source:HGNC Symbol;Acc:HGNC:5173] |
| IDH1 | isocitrate dehydrogenase (NADP(+)) 1 [Source:HGNC Symbol;Acc:HGNC:5382] |
| IDH2 | isocitrate dehydrogenase (NADP(+)) 2 [Source:HGNC Symbol;Acc:HGNC:5383] |
| IKZF1 | IKAROS family zinc finger 1 [Source:HGNC Symbol;Acc:HGNC:13176] |
| IRF4 | interferon regulatory factor 4 [Source:HGNC Symbol;Acc:HGNC:6119] |
| JAK2 | Janus kinase 2 [Source:HGNC Symbol;Acc:HGNC:6192] |
| JAK3 | Janus kinase 3 [Source:HGNC Symbol;Acc:HGNC:6193] |
| KIT | KIT proto-oncogene, receptor tyrosine kinase [Source:HGNC Symbol;Acc:HGNC:6342] |
| KMT2A | lysine methyltransferase 2A [Source:HGNC Symbol;Acc:HGNC:7132] |
| KMT2D | lysine methyltransferase 2D [Source:HGNC Symbol;Acc:HGNC:7133] |
| KRAS | KRAS proto-oncogene, GTPase [Source:HGNC Symbol;Acc:HGNC:6407] |
| LTB | lymphotoxin beta [Source:HGNC Symbol;Acc:HGNC:6711] |
| MAP2K1 | mitogen-activated protein kinase kinase 1 [Source:HGNC Symbol;Acc:HGNC:6840] |
| MAP2K2 | mitogen-activated protein kinase kinase 2 [Source:HGNC Symbol;Acc:HGNC:6842] |
| MEF2B | myocyte enhancer factor 2B [Source:HGNC Symbol;Acc:HGNC:6995] |
| MET | MET proto-oncogene, receptor tyrosine kinase [Source:HGNC Symbol;Acc:HGNC:7029] |
| MLH1 | mutL homolog 1 [Source:HGNC Symbol;Acc:HGNC:7127] |
| MPL | MPL proto-oncogene, thrombopoietin receptor [Source:HGNC Symbol;Acc:HGNC:7217] |
| MRE11 | MRE11 homolog, double strand break repair nuclease [Source:HGNC Symbol;Acc:HGNC:7230] |
| MSH2 | mutS homolog 2 [Source:HGNC Symbol;Acc:HGNC:7325] |
| MSH6 | mutS homolog 6 [Source:HGNC Symbol;Acc:HGNC:7329] |
| MTOR | mechanistic target of rapamycin kinase [Source:HGNC Symbol;Acc:HGNC:3942] |
| MUTYH | mutY DNA glycosylase [Source:HGNC Symbol;Acc:HGNC:7527] |
| MYD88 | MYD88 innate immune signal transduction adaptor [Source:HGNC Symbol;Acc:HGNC:7562] |
| MYOD1 | myogenic differentiation 1 [Source:HGNC Symbol;Acc:HGNC:7611] |
| NBN | nibrin [Source:HGNC Symbol;Acc:HGNC:7652] |
| NF1 | neurofibromin 1 [Source:HGNC Symbol;Acc:HGNC:7765] |
| NF2 | neurofibromin 2 [Source:HGNC Symbol;Acc:HGNC:7773] |
| NOTCH1 | notch receptor 1 [Source:HGNC Symbol;Acc:HGNC:7881] |
| NPM1 | nucleophosmin 1 [Source:HGNC Symbol;Acc:HGNC:7910] |
| NRAS | NRAS proto-oncogene, GTPase [Source:HGNC Symbol;Acc:HGNC:7989] |
| NTRK1 | neurotrophic receptor tyrosine kinase 1 [Source:HGNC Symbol;Acc:HGNC:8031] |
| NTRK2 | neurotrophic receptor tyrosine kinase 2 [Source:HGNC Symbol;Acc:HGNC:8032] |
| NTRK3 | neurotrophic receptor tyrosine kinase 3 [Source:HGNC Symbol;Acc:HGNC:8033] |
| NUTM1 | NUT midline carcinoma family member 1 [Source:HGNC Symbol;Acc:HGNC:29919] |
| PALB2 | partner and localizer of BRCA2 [Source:HGNC Symbol;Acc:HGNC:26144] |
| PAX8 | paired box 8 [Source:HGNC Symbol;Acc:HGNC:8622] |
| PDGFRA | platelet derived growth factor receptor alpha [Source:HGNC Symbol;Acc:HGNC:8803] |
| PDGFRB | platelet derived growth factor receptor beta [Source:HGNC Symbol;Acc:HGNC:8804] |
| PIK3CA | phosphatidylinositol-4,5-bisphosphate 3-kinase catalytic subunit alpha [Source:HGNC Symbol;Acc:HGNC:8975] |
| PIK3R1 | phosphoinositide-3-kinase regulatory subunit 1 [Source:HGNC Symbol;Acc:HGNC:8979] |
| PMS2 | PMS1 homolog 2, mismatch repair system component [Source:HGNC Symbol;Acc:HGNC:9122] |
| POLD1 | DNA polymerase delta 1, catalytic subunit [Source:HGNC Symbol;Acc:HGNC:9175] |
| POLE | DNA polymerase epsilon, catalytic subunit [Source:HGNC Symbol;Acc:HGNC:9177] |
| PPM1D | protein phosphatase, Mg2+/Mn2+ dependent 1D [Source:HGNC Symbol;Acc:HGNC:9277] |
| PRKAR1A | protein kinase cAMP-dependent type I regulatory subunit alpha [Source:HGNC Symbol;Acc:HGNC:9388] |
| PTEN | phosphatase and tensin homolog [Source:HGNC Symbol;Acc:HGNC:9588] |
| PTPN11 | protein tyrosine phosphatase non-receptor type 11 [Source:HGNC Symbol;Acc:HGNC:9644] |
| RAD50 | RAD50 double strand break repair protein [Source:HGNC Symbol;Acc:HGNC:9816] |
| RAD51B | RAD51 paralog B [Source:HGNC Symbol;Acc:HGNC:9822] |
| RAD51C | RAD51 paralog C [Source:HGNC Symbol;Acc:HGNC:9820] |
| RAD51D | RAD51 paralog D [Source:HGNC Symbol;Acc:HGNC:9823] |
| RAD54L | RAD54 like [Source:HGNC Symbol;Acc:HGNC:9826] |
| RASAL1 | RAS protein activator like 1 [Source:HGNC Symbol;Acc:HGNC:9873] |
| RB1 | RB transcriptional corepressor 1 [Source:HGNC Symbol;Acc:HGNC:9884] |
| RET | ret proto-oncogene [Source:HGNC Symbol;Acc:HGNC:9967] |
| RHOA | ras homolog family member A [Source:HGNC Symbol;Acc:HGNC:667] |
| RICTOR | RPTOR independent companion of MTOR complex 2 [Source:HGNC Symbol;Acc:HGNC:28611] |
| ROS1 | ROS proto-oncogene 1, receptor tyrosine kinase [Source:HGNC Symbol;Acc:HGNC:10261] |
| RUNX1 | RUNX family transcription factor 1 [Source:HGNC Symbol;Acc:HGNC:10471] |
| SETBP1 | SET binding protein 1 [Source:HGNC Symbol;Acc:HGNC:15573] |
| SF3B1 | splicing factor 3b subunit 1 [Source:HGNC Symbol;Acc:HGNC:10768] |
| SMAD4 | SMAD family member 4 [Source:HGNC Symbol;Acc:HGNC:6770] |
| SMARCA4 | SWI/SNF related, matrix associated, actin dependent regulator of chromatin, subfamily a, member 4 [Source:HGNC Symbol;Acc:HGNC:11100] |
| SMARCB1 | SWI/SNF related, matrix associated, actin dependent regulator of chromatin, subfamily b, member 1 [Source:HGNC Symbol;Acc:HGNC:11103] |
| SMO | smoothened, frizzled class receptor [Source:HGNC Symbol;Acc:HGNC:11119] |
| SRSF2 | serine and arginine rich splicing factor 2 [Source:HGNC Symbol;Acc:HGNC:10783] |
| STAG2 | stromal antigen 2 [Source:HGNC Symbol;Acc:HGNC:11355] |
| STAT3 | signal transducer and activator of transcription 3 [Source:HGNC Symbol;Acc:HGNC:11364] |
| STK11 | serine/threonine kinase 11 [Source:HGNC Symbol;Acc:HGNC:11389] |
| TENT5C | terminal nucleotidyltransferase 5C [Source:HGNC Symbol;Acc:HGNC:24712] |
| TERT | telomerase reverse transcriptase [Source:HGNC Symbol;Acc:HGNC:11730] |
| TET2 | tet methylcytosine dioxygenase 2 [Source:HGNC Symbol;Acc:HGNC:25941] |
| TNFAIP3 | TNF alpha induced protein 3 [Source:HGNC Symbol;Acc:HGNC:11896] |
| TNFRSF14 | TNF receptor superfamily member 14 [Source:HGNC Symbol;Acc:HGNC:11912] |
| TP53 | tumor protein p53 [Source:HGNC Symbol;Acc:HGNC:11998] |
| TRAF3 | TNF receptor associated factor 3 [Source:HGNC Symbol;Acc:HGNC:12033] |
| TSC1 | TSC complex subunit 1 [Source:HGNC Symbol;Acc:HGNC:12362] |
| TSC2 | TSC complex subunit 2 [Source:HGNC Symbol;Acc:HGNC:12363] |
| U2AF1 | U2 small nuclear RNA auxiliary factor 1 [Source:HGNC Symbol;Acc:HGNC:12453] |
| VAV1 | vav guanine nucleotide exchange factor 1 [Source:HGNC Symbol;Acc:HGNC:12657] |
| VHL | von Hippel-Lindau tumor suppressor [Source:HGNC Symbol;Acc:HGNC:12687] |
| WT1 | WT1 transcription factor [Source:HGNC Symbol;Acc:HGNC:12796] |
| XRCC2 | X-ray repair cross complementing 2 [Source:HGNC Symbol;Acc:HGNC:12829] |
| ZRSR2 | zinc finger CCCH-type, RNA binding motif and serine/arginine rich 2 [Source:HGNC Symbol;Acc:HGNC:23019] |
